# Supplementary material for: A natural experiment study: Low-profile double plating versus single plating techniques in midshaft clavicle fractures—Study protocol
Source: PLoS One. 2023 Sep 8;18(9):e0291238. doi: 10.1371/journal.pone.0291238 (PMC10490911; doi:10.1371/journal.pone.0291238)
Supplement: S4 File — (PDF) [file pone.0291238.s004.pdf]

SINGLE VS DOUBLE PLATING FOR MIDSHAFT CLAVICLE FRACTURES INCLUSION FORMS

Einschluss
Operativ mit Plattenosteosynthese therapierte Claviculaschaftfrakturen (single/double plate)

Ausschluss
OP >14 Tage posttraumatisch
Offene, pathologische oder Refraktur
Follow-up unmöglich (hochgradige kognitive Einschränkung, Wohnsitz im Ausland, etc)

Kein Ausschluss (neu)
Begleitverletzungen

To Do

- **Unterschrift <u>consent 2x</u> (Seiten 7 & 8)**
- **Fragebogen vollständig ausfüllen lassen**
- **Zurück ins Fächli (Assibüro 8. Stock) oder per mail**

Bitte jeweils kurze Rückmeldung per mail oder Tel

Operative Technik zur Versorgung eines Bruchs des Schlüsselbeinschafts im mittleren Drittel

Studie zum Vergleich der operativen Versorgung eines Bruchs des Schlüsselbeinschafts im mittleren Drittel mittels Doppelplatte versus Einzelplatte

Sehr geehrte Dame, sehr geehrter Herr

Wir fragen Sie hier an, ob Sie bereit wären, an unserem Forschungsvorhaben mitzuwirken.

Ihre Teilnahme ist freiwillig. Alle Daten, die in diesem Forschungsprojekt erhoben werden, unterliegen strengen Datenschutzvorschriften.

Diese Studie wird organisiert durch:

Principle Investigator:
PD Dr. Frank Beeres, Co-Chefarzt Klinik für Orthopädie und Unfallchirurgie

Co-Investigator:
Yannic Lecoultre, Assistenzarzt Klinik für Orthopädie und Unfallchirurgie

In einem Gespräch erklären wir Ihnen die wichtigsten Punkte und beantworten Ihre Fragen. Damit Sie sich bereits jetzt ein Bild machen können, hier das Wichtigste vorweg. Im Anschluss folgen dann weitere, detaillierte Informationen.

Warum führen wir dieses Forschungsvorhaben durch?

- Bei Brüchen des Schlüsselbeins des mittleren Drittels bei erwachsenen Patienten ist es aktuell noch umstritten welche operative Technik überlegen ist.
- In unserem Forschungsvorhaben wollen wir herausfinden, ob eine Technik der anderen überlegen ist.

Was muss ich bei einer Teilnahme tun? – Was geschieht mit mir bei einer Teilnahme?

- Form der Teilnahme: Wenn Sie sich entscheiden mitzumachen, ändert sich an der bereits geplanten Behandlung nichts.
- Ablauf der Teilnahme: Wenn Sie teilnehmen, werden wir sie zu unterschiedlichen Zeitpunkten (im Rahmen der geplanten Nachkontrollen) bitten, einige Fragebögen auszufüllen. Das Ausfüllen der Fragebögen wird maximal 15 Minuten in Anspruch nehmen.

Welcher Nutzen und welches Risiko sind damit verbunden?

- **Nutzen**

- Sie haben keinen direkten Nutzen, wenn Sie bei diesem Forschungsvorhaben mitmachen.
- Sie helfen mit Ihrer Teilnahme künftigen Patientinnen und Patienten.

- **Risiko und Belastung**
  - Sie sind keinem zusätzlichen Risiko ausgesetzt. Die Belastung begrenzt sich auf einen geringen zeitlichen Aufwand, um die Fragebögen auszufüllen.

Mit Ihrer Unterschrift am Ende des Dokuments bezeugen Sie, dass Sie freiwillig teilnehmen und dass Sie die Inhalte dieses Dokuments verstanden haben.

Detaillierte Information

1. Ziel und Auswahl
Unser Forschungsvorhaben bezeichnen wir in dieser Informationsschrift als *Forschungsprojekt.* Wenn Sie an diesem Forschungsprojekt teilnehmen, sind Sie eine *Teilnehmerin* bzw. ein *Teilnehmer*.

In diesem Forschungsprojekt wollen wir untersuchen, ob die Versorgung des Bruchs mit Doppelplatte im Falle eines Bruchs des Schlüsselbeins im mittleren Drittel bei erwachsenen Patienten der Versorgung mit nur einer einzelnen Platte überlegen ist. Wir fragen Sie an, da alle Personen teilnehmen können, die mind. 16 Jahre alt sind und einen frischen Bruch des mittleren Drittels des Schlüsselbeins haben, welcher operativ in unserem Spital behandelt wird.

2. Allgemeine Informationen
Brüche des Schlüsselbeinschafts im mittleren Drittel sind Verletzungen, welche oft aktive und junge Patienten betreffen. Trotz ihrer relativen Häufigkeit von 2-5% aller Brüche wird die optimale Behandlung solcher Brüche bei erwachsenen Patienten noch immer diskutiert. Bisherige Studien konnten diese Frage nicht ausreichen beantworten. Aus diesem Grund möchten wir herausfinden, ob eine Behandlungsform der anderen überlegen ist.
In diesem internationalen, multizentrischen Forschungsprojekt werden wir die operative Behandlung mittels zweier kleiner Platten mit der Behandlung mittels dickerer Einzelplatte vergleichen. Wenn Sie bei diesem Forschungsprojekt teilnehmen, ändert sich an Ihrer Behandlung nichts. Die Entscheidung, welche der beiden Techniken angewandt wird, liegt weiterhin bei Ihrem behandelnden Arzt. Wir werden gewisse Informationen aus Ihrer Patientenakte entnehmen (Röntgenbilder, Alter, Geschlecht, Vorerkrankungen, genaue Therapie) und Sie während des Spitalaufenthaltes wie auch während den üblichen Nachkontrollen (nach sechs Wochen, drei Monaten und einem Jahr) bitten, Fragebögen auszufüllen. Zusätzlich werden wir Sie nach zwei Jahren telefonisch kontaktieren und wieder bitten Fragebögen auszufüllen. Sämtliche Informationen werden vertraulich behandelt und für das Forschungsprojekt verschlüsselt. Den Zeitaufwand für das Ausfüllen der Fragebögen schätzen wir auf 5-15 Minuten.
Um eine gute Aussagekraft zu erhalten müssen rund 100 Patienten an dem Projekt teilnehmen. Um diese Zahl innert nützlicher Frist zu erreichen, wird das Projekt gemeinsam mit den anderen LUKS Standorten, dem Kantonsspital Obwalden wie auch drei Spitälern in den Niederlanden durchgeführt.
Das Forschungsprojekt entspricht den gesetzlichen Vorgaben in der Schweiz. Ausserdem beachten wir die international anerkannten Richtlinien. Die zuständige Ethikkommission hat das Forschungsprojekt geprüft und bewilligt.

3. Ablauf
Zum Zeitpunkt der ersten Vorstellung im Spital oder im Rahmen der ersten Verlaufskontrolle werden wir Sie fragen, ob Sie teilnehmen möchten. Sofern Sie Ihre schriftliche Einwilligung erteilt haben, werden wir Ihnen einen Fragebogen zum Ausfüllen geben.
Die Therapie erfolgt wie von Ihrem behandelnden Arzt empfohlen. Das Forschungsprojekt beeinflusst diese Entscheidung nicht. Im Rahmen der üblichen Nachkontrollen (nach sechs Wochen, drei Monaten und einem Jahr) werden wir Sie bitten, einen Fragebogen auszufüllen (zeitlicher Mehraufwand von ca. 5-15 Minuten). Die routinemässig durchgeführten Röntgenbilder werden ebenfalls für das Projekt evaluiert. Zwei Jahre nach dem Unfall werden wir Sie telefonisch kontaktieren und mit Ihnen erneut einen Fragebogen durchgehen. Ein zusätzlicher Klinikbesuch ist aufgrund der Studie nicht notwendig.

Es kann sein, dass wir Sie von dem Forschungsprojekt vorzeitig ausschliessen müssen. Dies würde Ihre Behandlung in keiner Weise beeinflussen.

4. Nutzen

Sie werden persönlich keinen Nutzen von der Teilnahme haben. Die Resultate der Studie können aber zu einer Optimierung der Behandlung von zukünftigen Patienten mit solchen Verletzungen führen.

5. Freiwilligkeit und Pflichten

Sie nehmen freiwillig teil. Wenn Sie nicht an diesem Forschungsprojekt teilnehmen oder später Ihre Teilnahme zurückziehen wollen, müssen Sie dies nicht begründen. Ihre Behandlung/Betreuung ist unabhängig von Ihrem Entscheid gewährleistet.

Wenn Sie an diesem Forschungsprojekt teilnehmen, werden Sie gebeten:

- sich an die Vorgaben und Anforderungen des Forschungsprojekts durch den Prüfplan zu halten (z.B. Sprechstundentermine wahrnehmen)
- Ihre Prüfärztin/Ihren Prüfarzt über den Verlauf der Erkrankung zu informieren und neue Symptome, neue Beschwerden und Änderungen im Befinden zu melden
- Ihre Prüfärztin/Ihren Prüfarzt über die gleichzeitige Behandlung und Therapie bei anderen Ärztinnen und Ärzten zu informieren.

6. Risiken und Belastungen

Es müssen Fragebögen im Rahmen der Nachkontrollen und zwei Jahre nach Unfall im Rahmen eines Telefongespräches ausgefüllt werden. Bis auf einen gewissen zeitlichen Aufwand sind Sie keinen Risiken oder Belastungen ausgesetzt.

7. Ergebnisse

Es gibt

1. Individuelle Ergebnisse des Forschungsprojekts, die Sie direkt betreffen,
2. Objektive End-Ergebnisse des gesamten Forschungsprojekts.

Zu 1: Die Prüfperson wird Sie im Verlauf des Projekts über allfällige für Sie persönlich wichtige, neue Ergebnisse und Erkenntnisse informieren. Sie werden mündlich und schriftlich informiert und können dann erneut entscheiden, ob Sie an dem Projekt weiter teilnehmen möchten.

Zu 2: Ihre Prüfärztin/Ihr Prüfarzt kann Ihnen am Ende des Forschungsprojekts eine Zusammenfassung der Gesamtergebnisse zukommen lassen.

8. Vertraulichkeit von Daten und Proben

8.1. Datenverarbeitung und Verschlüsselung

Für dieses Forschungsprojekt werden Daten zu Ihrer Person und Gesundheit erfasst und bearbeitet, teilweise in automatisierter Form. Bei der Datenerhebung werden Ihre Daten verschlüsselt. Verschlüsselung bedeutet, dass alle Bezugsdaten, die Sie identifizieren könnten (Name, Geburtsdatum etc.), gelöscht und durch einen Code ersetzt werden. Personen, die keinen Zugang zu dieser Schlüssel-Liste haben, können keine Rückschlüsse auf Ihre Person ziehen. Die Schlüssel-Liste bleibt immer passwortgeschützt auf dem Server des Luzerner Kantonsspitals.
Nur sehr wenige Fachpersonen werden Ihre unverschlüsselten Daten sehen und zwar nur, um Aufgaben im Rahmen des Forschungsprojekts zu erfüllen. Diese Personen unterliegen der Schweigepflicht. Sie als teilnehmende Person haben das Recht auf Einsicht in Ihre Daten.

8.2. Datenschutz

Alle Vorgaben des Datenschutzes werden eingehalten. Es ist möglich, dass Ihre Daten in verschlüsselter Form, zum Beispiel für eine Publikation, übermittelt werden müssen und anderen Forschern zur Verfügung gestellt werden können. Wenn gesundheitsbezogene Daten vor Ort gelagert werden, handelt es sich um eine Datenbank für Forschungszwecke.

Ärztinnen und Ärzte, die für die Nachbehandlung verantwortlich sind, können kontaktiert werden, um Auskunft über Ihren Gesundheitszustand zu geben.

- **8.3. Datenschutz bei Weiterverwendung**

Ihre Daten könnten für die Beantwortung von anderen Fragestellungen zu einem späteren Zeitpunkt wichtig sein und in eine andere Datenbank integriert und wieder verwendet werden. Diese andere Datenbank muss die gleichen Standards einhalten wie die Datenbank zu diesem Projekt.
Für diese Weiterverwendung bitten wir Sie, ganz am Ende dieses Dokuments eine weitere Einwilligungserklärung zu unterzeichnen. Diese zweite Einwilligung ist unabhängig von der Teilnahme an diesem Projekt.

8.4. Einsichtsrechte bei Kontrollen

Dieses Forschungsprojekt kann durch die zuständige Ethikkommission und durch die Projektleitung überprüft werden. Die Prüfärztin/der Prüfarzt muss dann Ihre Daten für solche Kontrollen offenlegen. Alle müssen absolute Vertraulichkeit wahren.

9. Rücktritt

Sie können jederzeit von dem Forschungsprojekt zurücktreten. Die bis dahin erhobenen Daten werden in diesem Fall allerdings noch verschlüsselt ausgewertet. Im Falle eines Rücktritts bleiben Ihre Daten weiterhin verschlüsselt in den Projektdokumenten.

10. Entschädigung

Wenn Sie an diesem Forschungsprojekt teilnehmen, bekommen Sie dafür keine Entschädigung.
Es entstehen Ihnen oder Ihrer Krankenkasse keine Kosten durch die Teilnahme.

11. Haftung

Falls Sie durch das Forschungsprojekt einen Schaden erleiden sollten, haftet die Institution (Luzerner Kantonsspital resp. Kantonsspital Obwalden), die das Forschungsprojekt veranlasst hat und für die Durchführung verantwortlich ist. Die Voraussetzungen und das Vorgehen sind gesetzlich geregelt. Wenn Sie einen Schaden erlitten haben, so wenden Sie sich bitte an die Prüfärztin/den Prüfarzt.

12. Finanzierung

Das Forschungsprojekt wird durch das Luzerner Kantonsspital finanziert.

13. Kontaktperson(en)

Sie dürfen jederzeit Fragen zur Projektteilnahme stellen. Auch bei Unsicherheiten, die während des Forschungsprojekts oder danach auftreten, wenden Sie sich bitte an:

- Yannic Lecoultre
  Assistenzarzt Klinik für Orthopäedie und Unfallchirurgie, Luzerner Kantonsspital

- PD Dr. Frank Beeres
  Co-Chefarzt Klinik für Orthopädie und Unfallchirurgie, Luzerner Kantonsspital

Einwilligungserklärung

Schriftliche Einwilligungserklärung zur Teilnahme an einem Forschungsprojekt
Bitte lesen Sie dieses Formular sorgfältig durch. Bitte fragen Sie nach, wenn Sie etwas nicht verstehen oder wissen möchten. Für die Teilnahme ist Ihre schriftliche Einwilligung notwendig.

**BASEC-Nummer (nach Einreichung):**	2022-00574
**Titel des Forschungsprojekts (wissenschaftlich und Laiensprache)**:	Double Plating versus single plating techniques in midshaft clavicle fractures in adult patients<br><br>Versorgung eines Bruchs des Schlüsselbeinschafts im mittleren Drittel mittels Doppelplatten- vs. Einzelplatten bei Patienten im Erwachsenenalter
**Verantwortliche Institution (Projektleitung mit Adresse)**:	PD Dr Frank Beeres<br><br>Spitalstrasse<br>6000 Luzern
**Ort der Durchführung**:	Luzerner Kantonsspital
**Leiterin/Leiter des Forschungsprojekts am Studienort:**	Yannic Lecoultre<br>Assistenzarzt Klinik für Orthopädie und Unfallchirurgie<br>Luzerner Kantonsspital
**Teilnehmerin/Teilnehmer:**<br>Name und Vorname in Druckbuchstaben:<br>Geburtsdatum:	

- Ich wurde von der unterzeichnenden Prüfärztin/dem unterzeichnenden Prüfarzt mündlich und schriftlich über den Zweck, den Ablauf des Forschungsprojekts, über mögliche Vor- und Nachteile sowie über eventuelle Risiken informiert.
- Ich nehme an diesem Forschungsprojekt freiwillig teil und akzeptiere den Inhalt der zum oben genannten Forschungsprojekt abgegebenen schriftlichen Information. Ich hatte genügend Zeit, meine Entscheidung zu treffen.
- Meine Fragen im Zusammenhang mit der Teilnahme an diesem Forschungsprojekt sind mir beantwortet worden. Ich behalte die schriftliche Information und erhalte eine Kopie meiner schriftlichen Einwilligungserklärung.
- Ich bin einverstanden, dass die zuständigen Fachleute der Projektleitung und der für dieses Forschungsprojekt zuständigen Ethikkommission zu Prüf- und Kontrollzwecken in meine unverschlüsselten Daten Einsicht nehmen dürfen, jedoch unter strikter Einhaltung der Vertraulichkeit.
- Bei Ergebnissen, die direkt meine Gesundheit betreffen, werde ich informiert. Wenn ich das nicht wünsche, informiere ich meine Prüfärztin/meinen Prüfarzt.
- Ich weiss, dass meine gesundheitsbezogenen und persönlichen Daten nur in verschlüsselter Form zu Forschungszwecken für dieses Forschungsprojekt weitergegeben werden können (auch ins Ausland). Der Sponsor gewährleistet, dass der Datenschutz nach Schweizer Standard eingehalten wird.
- Ich kann jederzeit und ohne Angabe von Gründen von der Teilnahme zurücktreten. Meine weitere Behandlung ist unabhängig von der Teilnahme am Forschungsprojekt gewährleistet. Die bis dahin erhobenen Daten und Proben werden für die Auswertung des Forschungsprojekts noch verwendet.
- Ich bin einverstanden, dass meine Hausärztin/mein Hausarzt über meine Teilnahme an dem Forschungsprojekt informiert wird.

- Die Institution (Luzerner Kantonsspital) haftet für allfällige Schäden.

Ort, Datum	Unterschrift Teilnehmerin/Teilnehmer

Bestätigung der Prüfärztin/des Prüfarztes/der Prüfperson: Hiermit bestätige ich, dass ich dieser Teilnehmerin/diesem Teilnehmer Wesen, Bedeutung und Tragweite des Forschungsprojekts erläutert habe. Ich versichere, alle im Zusammenhang mit diesem Forschungsprojekt stehenden Verpflichtungen gemäss in der Schweiz geltenden Rechts zu erfüllen. Sollte ich im Verlauf des Forschungsprojekts von Aspekten erfahren, welche die Bereitschaft der Teilnehmerin/des Teilnehmers an dem Forschungsprojekt beeinflussen könnten, werde ich sie/ihn umgehend darüber informieren.

Ort, Datum	Name und Vorname der Prüfperson in Druckbuchstaben<br><br>Unterschrift der Prüfperson

Einwilligungserklärung für Weiterverwendung von Daten in verschlüsselter Form

**BASEC-Nummer (nach Einreichung):**	2022-00574
**Titel des Forschungsprojekts (wissenschaftlich und Laiensprache):**	Double Plating versus single plating techniques in midshaft clavicle fractures in adult patients<br><br>Versorgung eines Bruchs des Schlüsselbeinschafts im mittleren Drittel mittels Doppelplatten- vs. Einzelplatten bei Patienten im Erwachsenenalter
**Teilnehmerin/Teilnehmer:**<br>Name und Vorname in Druckbuchstaben:<br>Geburtsdatum:	

Ich erlaube, dass meine verschlüsselten Daten aus diesem Forschungsprojekt für die medizinische Forschung weiterverwendet werden dürfen.
Ich habe verstanden, dass die Daten verschlüsselt sind und der Schüssel sicher aufbewahrt wird. Die Daten können im In- und Ausland an andere Datenbanken zur Analyse gesendet werden, wenn diese dieselben Standards wie in der Schweiz einhalten. Alle rechtlichen Vorgaben zum Datenschutz werden eingehalten.

Ich entscheide freiwillig und kann diesen Entscheid zu jedem Zeitpunkt wieder zurücknehmen. Wenn ich zurücktrete, werden meine Daten anonymisiert. Ich informiere lediglich meine Prüfärztin/meinen Prüfarzt/die Projektleitung und muss diesen Entscheid nicht begründen.

Normalerweise werden alle Daten gesamthaft ausgewertet und die Ergebnisse zusammenfassend publiziert. Sollte sich ein für meine Gesundheit wichtiges Ergebnis ergeben, ist es möglich, dass ich kontaktiert werde. Wenn ich das nicht wünsche, teile ich es meiner Prüfärztin/meinem Prüfarzt mit.

Ort, Datum	Unterschrift Teilnehmerin/Teilnehmer

Bestätigung der Prüfärztin/des Prüfarztes/der Prüfperson: Hiermit bestätige ich, dass ich dieser Teilnehmerin/diesem Teilnehmerin Wesen, Bedeutung und Tragweite der Weiterverwendung von Proben und/oder genetischen Daten erläutert habe.

Ort, Datum	Name und Vorname der Prüfperson in Druckbuchstaben<br><br>Unterschrift der Prüfperson

ANLEITUNG

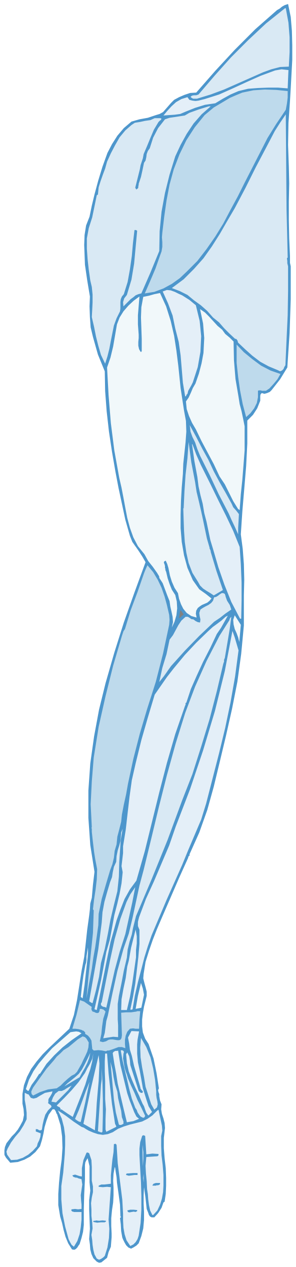

Dieser Fragebogen beschäftigt sich sowohl mit Ihren Beschwerden, als auch mit Ihren Fähigkeiten, bestimmte Tätigkeiten auszuführen.

Bitte beantworten *Sie alle Fragen* gemäß Ihrem Zustand in der Woche **vor Ihrem Unfall**, indem Sie einfach die entsprechende Zahl ankreuzen.

Wenn Sie in der vergangenen Woche keine Gelegenheit hatten, eine der unten aufgeführten Tätigkeiten durchzuführen, so wählen Sie die Antwort aus, die Ihrer Meinung nach *am ehesten* zutreffen würde.

Es ist nicht entscheidend, mit welchem Arm oder welcher Hand Sie diese Tätigkeiten ausüben. Antworten Sie Ihrer Fähigkeit entsprechend, ungeachtet, wie Sie die Aufgaben durchführen konnten.

© Institute for Work & Health 2006. All rights reserved.

Deutsche Version: Günter Germann, Angela Harth, Gerhard Wind, Erhan Demir.

*Quick*DASH

Bitte schätzen Sie Ihre Fähigkeit ein, wie Sie folgende Tätigkeiten in der Woche vor Ihrem Unfall durchgeführt haben, indem Sie die entsprechende Zahl ankreuzen.

	Keine Schwierigkeiten	Geringe Schwierigkeiten	Mäßige Schwierigkeiten	Erhebliche Schwierigkeiten	Nicht möglich
1. Ein neues oder festverschlossenes Glas öffnen	1	2	3	4	5
2. Schwere Hausarbeit (z. B Wände abwaschen, Boden putzen)	1	2	3	4	5
3. Eine Einkaufstasche oder einen Aktenkoffer tragen	1	2	3	4	5
4. Ihren Rücken waschen	1	2	3	4	5
5. Ein Messer benutzen, um Lebensmittel zu schneiden	1	2	3	4	5
6. Freizeitaktivitäten, bei denen auf Ihren Arm, Schulter oder Hand Druck oder Stoß ausgeübt wird (z.B. Golf, Hämmern, Tennis, usw.)	1	2	3	4	5

	Überhaupt nicht	Ein wenig	Mäßig	Ziemlich	Sehr
7. In welchem Ausmaß haben Ihre Schulter-, Arm- oder Handprobleme Ihre normalen sozialen Aktivitäten mit Familie, Freunden, Nachbarn oder anderen Gruppen während der vergangenen Woche beeinträchtigt? *(Bitte kreuzen Sie die entsprechende Zahl an)*	1	2	3	4	5

	Überhaupt nicht eingeschränkt	Ein wenig eingeschränkt	Mäßig eingeschränkt	Sehr eingeschränkt	Nicht möglich
8. Waren Sie in der Woche **vor Ihrem Unfall** durch Ihre Schulter-, Arm- oder Handprobleme in Ihrer Arbeit oder anderen alltäglichen Aktivitäten eingeschränkt? *(Bitte kreuzen Sie die entsprechende Zahl an)*	1	2	3	4	5

Bitte schätzen Sie die Schwere der folgenden Symptome während der Woche vor Ihrem Unfall ein. *(Bitte kreuzen Sie in jeder Zeile die entsprechende Zahl an)*

	Keine	Leichte	Mäßige	Starke	Sehr starke
9. Schmerzen in Schulter, Arm oder Hand	**1**	**2**	**3**	**4**	**5**
10. Kribbeln (Nadelstiche) in Schulter, Arm oder Hand	**1**	**2**	**3**	**4**	**5**

	Keine Schwierigkeiten	Geringe Schwierigkeiten	Mäßige Schwierigkeiten	Erhebliche Schwierigkeiten	Nicht möglich
11. Wie groß waren Ihre Schlafstörungen in der letzten Woche aufgrund von Schmerzen im Schulter-, Arm- oder Handbereich? *(Bitte kreuzen Sie die entsprechende Zahl an)*	**1**	**2**	**3**	**4**	**5**

*Quick*DASH

ARBEITS- UND BERUFS-MODUL (OPTIONAL)

Die folgenden Fragen beziehen sich auf den Einfluss Ihres Schulter-, Arm- oder Handproblems auf Ihre Arbeit (einschließlich Haushaltsführung, falls dies Ihre Hauptbeschäftigung ist).

Bitte geben Sie Ihre/n Arbeit/Beruf hier an: ____________________

□ Ich bin nicht berufstätig (Sie können diesen Bereich auslassen).

Bitte kreuzen Sie die Zahl an, die Ihre körperlichen Fähigkeiten in der Woche **vor Ihrem Unfall** am besten beschreibt.

**Hatten Sie irgendwelche Schwierigkeiten:**	Keine Schwierigkeiten	Geringe Schwierigkeiten	Mäßige Schwierigkeiten	Erhebliche Schwierigkeiten	Nicht möglich
12. In der üblichen Art und Weise zu arbeiten?	**1**	**2**	**3**	**4**	**5**
13. Aufgrund der Schmerzen in Schulter, Arm oder Hand Ihre übliche Arbeit zu erledigen?	**1**	**2**	**3**	**4**	**5**
14. So gut zu arbeiten wie Sie es möchten?	**1**	**2**	**3**	**4**	**5**
15. Die bisher gewohnte Zeit mit Ihrer Arbeit zu verbringen?	**1**	**2**	**3**	**4**	**5**

SPORT- UND MUSIK-MODUL (OPTIONAL)

Die folgenden Fragen beziehen sich auf den Einfluss Ihres Schulter-, Arm- oder Handproblems auf das Spielen Ihres Musikinstrumentes oder auf das Ausüben Ihres Sports oder auf beides. Wenn Sie mehr als ein Instrument spielen oder mehr als eine Sportart ausüben (oder beides), so beantworten Sie bitte die Fragen in bezug auf das Instrument oder die Sportart, die für Sie am wichtigsten ist.

Bitte geben Sie dieses Instrument bzw. diese Sportart hier an: ______________________________

□ Ich treibe keinen Sport und spiele kein Instrument (Sie können diesen Bereich auslassen).

Bitte kreuzen Sie die Zahl an, die Ihre körperlichen Fähigkeiten in der Woche vor Ihrem Unfall am besten beschreibt.

**Hatten Sie irgendwelche Schwierigkeiten:**	**Keine Schwierigkeiten**	**Geringe Schwierigkeiten**	**Mäßige Schwierigkeiten**	**Erhebliche Schwierigkeiten**	**Nicht möglich**
16. In der üblichen Art und Weise Ihr Musikinstrument zu spielen oder Sport zu treiben?	**1**	**2**	**3**	**4**	**5**
17. Aufgrund der Schmerzen in Schulter, Arm oder Hand Ihr Musikinstrument zu spielen oder Sport zu treiben?	**1**	**2**	**3**	**4**	**5**
18. So gut Ihr Musikinstrument zu spielen oder Sport zu treiben wie Sie es möchten?	**1**	**2**	**3**	**4**	**5**
19. Die bisher gewohnte Zeit mit dem Spielen Ihres Musikinstru-mentes oder mit Sporttreiben zu verbringen?	**1**	**2**	**3**	**4**	**5**

Auswertung der optionalen Module: Die Antwortpunkte der Fragen werden summiert; durch 4 (Anzahl der Fragen) dividiert; 1 wird subtrahiert und danach mit 25 multipliziert.

Für die Auswertung eines optionalen Moduls dürfen keine Antworten fehlen.

Bitte kreuzen Sie unter jeder Überschrift DAS Kästchen an, das Ihre Gesundheit **in der Woche vor Ihrem Unfall** am besten beschreibt.

14. BEWEGLICHKEIT / MOBILITÄT

Ich habe keine Probleme herumzugehen ❑
Ich habe leichte Probleme herumzugehen ❑
Ich habe mittelmässige Probleme herumzugehen ❑
Ich habe grosse Probleme herumzugehen ❑
Ich bin nicht in der Lage herumzugehen ❑

15. FÜR SICH SELBST SORGEN

Ich habe keine Probleme, mich selbst zu waschen oder anzuziehen ❑
Ich habe leichte Probleme, mich selbst zu waschen oder anzuziehen ❑
Ich habe mittelmässige Probleme, mich selbst zu waschen oder anzuziehen ❑
Ich habe grosse Probleme, mich selbst zu waschen oder anzuziehen ❑
Ich bin nicht in der Lage, mich selbst zu waschen oder anzuziehen ❑

16. ALLGEMEINE TÄTIGKEITEN *(z.B. Arbeit, Studium, Hausarbeit, Familien- oder Freizeitaktivitäten)*

Ich habe keine Probleme, meinen alltäglichen Tätigkeiten nachzugehen ❑
Ich habe leichte Probleme, meinen alltäglichen Tätigkeiten nachzugehen ❑
Ich habe mittelmässige Probleme, meinen alltäglichen Tätigkeiten nachzugehen ❑
Ich habe grosse Probleme, meinen alltäglichen Tätigkeiten nachzugehen ❑
Ich bin nicht in der Lage, meinen alltäglichen Tätigkeiten nachzugehen ❑

17. SCHMERZEN / KÖRPERLICHE BESCHWERDEN

Ich habe keine Schmerzen oder Beschwerden ❑
Ich habe leichte Schmerzen oder Beschwerden ❑
Ich habe mittelmässige Schmerzen oder Beschwerden ❑
Ich habe starke Schmerzen oder Beschwerden ❑
Ich habe extreme Schmerzen oder Beschwerden ❑

18. ANGST / NIEDERGESCHLAGENHEIT

Ich bin nicht ängstlich oder deprimiert ❑
Ich bin ein wenig ängstlich oder deprimiert ❑
Ich bin mittelmässig ängstlich oder deprimiert ❑
Ich bin sehr ängstlich oder deprimiert ❑
Ich bin extrem ängstlich oder deprimiert ❑

- Wir wollen herausfinden, wie gut oder schlecht Ihre Gesundheit **in der Woche vor Ihrem Unfall** war.

- Diese Skala ist mit Zahlen von 0 bis 100 versehen.

- 100 ist die beste Gesundheit, die Sie sich vorstellen können.
  0 (Null) ist die schlechteste Gesundheit, die Sie sich vorstellen können.

- Bitte kreuzen Sie den Punkt auf der Skala an, der Ihre Gesundheit in der Woche vor Ihrem Unfall am besten beschreibt.

- Jetzt tragen Sie bitte die Zahl, die Sie auf der Skala angekreuzt haben, in das Kästchen unten ein.

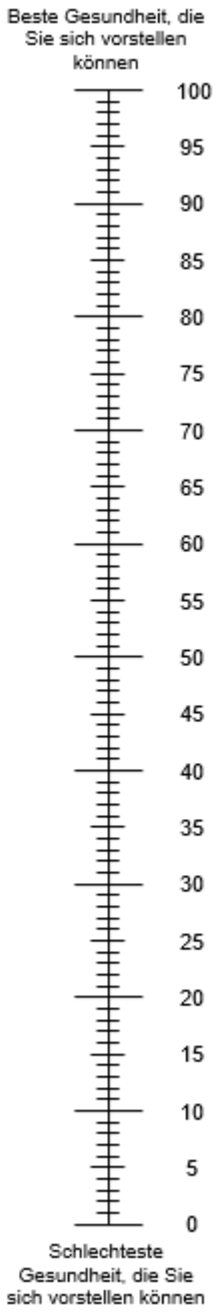

IHRE GESUNDHEIT IN DER WOCHE VOR IHREM UNFALL = ☐
