# Supplementary material for: A natural experiment study: Low-profile double plating versus single plating techniques in midshaft clavicle fractures—Study protocol
Source: PLoS One. 2023 Sep 8;18(9):e0291238. doi: 10.1371/journal.pone.0291238 (PMC10490911; doi:10.1371/journal.pone.0291238)
Supplement: S5 File — (PDF) [file pone.0291238.s005.pdf]

SINGLE VS DOUBLE PLATING FOR MIDSHAFT CLAVICLE FRACTURES INCLUSION FORMS

TRANSLATION (ORIGINAL IN GERMAN)

Inclusion
Surgically treated midshaft clavicle fractures (plate osteosynthesis)

Exclusion
OP >14 Tage post trauma
Open, pathologic or re-fracture
Follow-up impossible (cognitive impairment, residence abroad, etc.)

No exclusion
concomittant injuries

To Do

- **Signature <u>consent 2x</u> (page 7 & 8)**
- **Have questionnaire filled out completely**

Consent to participate in medical research:

Surgical technique for the treatment of a fracture of the shaft of the clavicle in the middle third

Comparison of treatment by single or double plating

Dear Madam, Dear Sir.

We are asking you to participate in our research project.

Your participation is voluntary. All data collected in this research project is subject to strict data protection regulations.

This study is organized by:
Principle Investigator:
PD Dr. Frank Beeres, Co-Chefarzt Klinik für Orthopädie und Unfallchirurgie

Co-Investigator:
Yannic Lecoultre, Assistenzarzt Klinik für Orthopädie und Unfallchirurgie

In a conversation we will explain the most important points and answer your questions. So that you can already get an idea, here are the most important points in advance. Further, detailed information will then follow.

Why are we conducting this research project?

- In midshaft clavicle fractures, the optimal standard of care is still subject to discussions.
- In our research project, we want to find out, if one technique is superior.

What do I have to do when I participate? - What happens to me when I participate?

- Form of participation: If you decide to participate, nothing will change in the already planned treatment.
- Procedure of participation: If you participate, we will ask you to fill in some questionnaires at different times (during the scheduled follow-up visits). Filling in the questionnaires will take a maximum of 15 minutes.

What are the associated benefits and risks?

- **Benefit**
  - You have no direct benefit by participating in this research project.
  - You will help future patients with your participation.
- **Risks and burden**
  - You are not exposed to any additional risk. The burden is limited to a small amount of time to complete the questionnaires.

By signing at the end of this document, you attest that you are participating voluntarily and that you understand the contents of this document.

Detailed information

1. Aim and selection
We refer to our research project as a research project in this handout. If you participate in this research project, you are a participant.

In this research project we want to investigate whether the treatment of the fracture with double plates is superior to the treatment with only a single plate in case of a fracture of the clavicle in the middle third in adult patients. We are asking you to participate because all persons can participate who are at least 16 years old and have a fresh fracture of the middle third of the clavicle, which is treated surgically in our hospital.

2. General information
Fractures of the middle third of the clavicle are injuries that often affect active and young patients. Despite their relative frequency of 2-5% of all fractures, the optimal treatment of such fractures in adult patients is still debated. Previous studies have not been able to adequately answer this question. For this reason, we would like to find out whether one treatment modality is superior to another.
In this international, multicenter research project, we will compare surgical treatment using two small plates with treatment using a thicker single plate. If you participate in this research project, your treatment will not change. It will still be up to your treating physician to decide which of the two techniques to use. We will take certain information from your patient file (X-rays, age, sex, previous diseases, exact therapy) and ask you to fill out questionnaires during your hospital stay as well as during the usual follow-up visits (after six weeks, three months and one year). In addition, we will contact you by phone after two years and ask you again to fill in questionnaires. All information will be kept confidential and encrypted for the research project. We estimate the time needed to complete the questionnaires to be 5-15 minutes.
In order to get a good result, about 100 patients have to participate in the project. In order to reach this number within a reasonable period of time, the project will be carried out together with the other LUKS sites, the Cantonal Hospital Obwalden as well as three hospitals in the Netherlands.
The research project complies with the legal requirements in Switzerland. We also comply with internationally recognized guidelines. The responsible ethics committee has reviewed and approved the research project.

3. Procedure
At the time of your first presentation at the hospital or during the first follow-up, we will ask you if you would like to participate. If you have given your written consent, we will give you a questionnaire to fill out.
The therapy will be as recommended by your treating physician. The research project does not influence this decision. As part of the usual follow-up visits (after six weeks, three months and one year), we will ask you to fill out a questionnaire (additional time of about 5-15 minutes). Routine x-rays will also be evaluated for the project. Two years after the accident, we will contact you by phone and go through a questionnaire with you again. An additional clinic visit is not necessary due to the study.
We may have to exclude you from the research project early. This would not affect your treatment in any way.

4. benefit
You will not personally benefit from participation. However, the results of the study may lead to an optimization of the treatment of future patients with such injuries.

2. Voluntariness and obligations
You participate voluntarily. If you do not wish to participate in this research project or later withdraw your participation, you do not have to justify this. Your treatment/care is guaranteed regardless of your decision.

If you participate in this research project, you are asked to:
to adhere to the guidelines and requirements of the research project through the protocol (e.g., attend office hours)
to keep your investigator informed about the course of the disease and to report new symptoms, new complaints and changes in well-being

to inform your investigator about concurrent treatment and therapy by other physicians.

3. Risks and burdens
Questionnaires must be completed during follow-up visits and two years after the accident during a telephone interview. Except for a certain amount of time, you are not exposed to any risks or burdens.

4. Results
There are
1. individual results of the research project that directly affect you,
2. objective final results of the whole research project.

Regarding 1: The investigator will inform you during the course of the project about any new results and findings that are important to you personally. You will be informed verbally and in writing and can then decide again whether you wish to continue participating in the project.

Re 2: Your investigator may send you a summary of the overall results at the end of the research project.

5 Confidentiality of data and samples
5.1 Data processing and encryption
For this research project, data about your person and health will be collected and processed, partly in automated form. During data collection, your data will be encrypted. Encryption means that all reference data that could identify you (name, date of birth, etc.) will be deleted and replaced by a code. People who do not have access to this key list cannot draw any conclusions about you. The key list always remains password-protected on the server of the Lucerne Cantonal Hospital.
Only very few specialists will see your unencrypted data and only to perform tasks within the research project. These persons are subject to the duty of confidentiality. You as a participating person have the right to see your data.

5.2 Data protection
All data protection regulations will be observed. It is possible that your data must be transmitted in encrypted form, for example for publication, and may be made available to other researchers. If health-related data is stored on site, it is a database for research purposes.
Physicians responsible for follow-up treatment may be contacted to provide information about your health status.

5.3 Data protection in case of further use
Your data could be important for answering other questions at a later time and could be integrated and reused in another database. This other database must comply with the same standards as the database for this project.
For this reuse, we ask you to sign another consent form at the very end of this document. This second consent is independent of your participation in this project.

5.4 Inspection rights in case of controls
This research project may be subject to inspection by the responsible ethics committee and by the project management. The investigator must then disclose your data for such inspections. All must maintain absolute confidentiality.

6 Withdrawal
You may withdraw from the research project at any time. In this case, however, the data collected up to that point will still be analyzed in encrypted form. In case of withdrawal, your data will remain encrypted in the project documents.

7. compensation
If you participate in this research project, you will not receive any compensation.
No costs will be incurred by you or your health insurance company as a result of your participation.

8 Liability

If you should suffer any damage as a result of the research project, the institution which initiated the research project is liable and is responsible for its implementation. The requirements and the procedure are regulated by law. If you have suffered damage, please contact the investigator.

9. Funding

The research project is funded by the LUKS

10. Contact details

You may ask questions about project participation at any time. Also, if you have any uncertainties that arise during the research project or afterwards, please contact:

- Yannic Lecoultre
  Assistenzarzt Klinik für Orthopäedie und Unfallchirurgie, Luzerner Kantonsspital
  e-mail: yannic.lecoultre@luks.ch, Tel 041 205 19 74

- PD Dr. Frank Beeres
  Co-Chefarzt Klinik für Orthopädie und Unfallchirurgie, Luzerner Kantonsspital

Declaration of consent

Written informed consent to participate in a research project.
Please read this form carefully. Please ask if there is anything you do not understand or would like to know. Your written consent is required for participation..

**BASEC-Nr**	2022-00574
**Title of the research project**	Double Plating versus single plating techniques in midshaft clavicle fractures in adult patients
**Responsible Institution (Project leader, Address)**:	PD Dr Frank Beeres<br><br>Spitalstrasse<br>6000 Luzern
**Location**	Luzerner Kantonsspital
**Local study coordinator**	Yannic Lecoultre<br>Assistenzarzt Klinik für Orthopädie und Unfallchirurgie<br>Luzerner Kantonsspital
**Participant**<br>Name and surname<br>Date of Birth	

- I have been informed verbally and in writing by the undersigned investigator about the purpose, the procedure of the research project, about possible advantages and disadvantages as well as about possible risks.
- I am voluntarily participating in this research project and accept the contents of the written information provided on the above research project. I have had sufficient time to make my decision.
- My questions related to participation in this research project have been answered. I am keeping the written information and receiving a copy of my written informed consent.
- I agree that the relevant experts of the project management and the ethics committee responsible for this research project may inspect my unencrypted data for review and control purposes, but under strict observance of confidentiality.
- In the case of results that directly affect my health, I will be informed. If I do not wish to be informed, I will inform my investigator.
- I know that my health-related and personal data can only be passed on in encrypted form for research purposes for this research project (also abroad). The sponsor ensures that data protection is maintained according to Swiss standards.
- I may withdraw from participation at any time and without giving reasons. My continued treatment is guaranteed regardless of participation in the research project. The data and samples collected up to that point will still be used for the evaluation of the research project.
- I agree that my family doctor will be informed about my participation in the research project.
- The institution (Luzerner Kantonsspital) is liable for any damages.

Location, date	Signature of the participant

Confirmation by the investigator: I hereby confirm that I have explained the nature, significance and scope of the research project to this participant. I assure that I will fulfill all obligations in connection with this research project in accordance with the law applicable in Switzerland. If, in the course of the research project, I learn of any aspects that could influence the participant's willingness to participate in the research project, I will inform him/her immediately.

Location, date	Name and surname of the investigator<br><br>Signature

Declaration of consent for further use of data in encrypted form

**BASEC-Number:**	2022-00574
**Title of the research project**	Double Plating versus single plating techniques in midshaft clavicle fractures in adult patients
**Participant**<br>Name and Surname	

I give permission for my encrypted data from this research project to be reused for medical research.
I understand that the data is encrypted and the key is kept secure. The data may be sent to other databases in Switzerland and abroad for analysis if they adhere to the same standards as in Switzerland. All legal requirements for data protection are complied with.

I decide voluntarily and can withdraw this decision at any time. If I withdraw, my data will be anonymized. I only inform my investigator/the project management and do not have to justify this decision.

Normally, all data are evaluated as a whole and the results are published in summary form. If there is a result that is important for my health, it is possible that I will be contacted. If I do not wish this, I will inform my investigator.

Location, date	Signature of the participant

Investigator/Investigator Acknowledgement: I hereby acknowledge that I have explained to this participant the nature, significance and implications of the further use of samples and/or genetic data.

Location, date	Name and surname of the investigator<br><br>Signature

DISABILITIES OF THE ARM, SHOULDER AND HAND

THE DASH

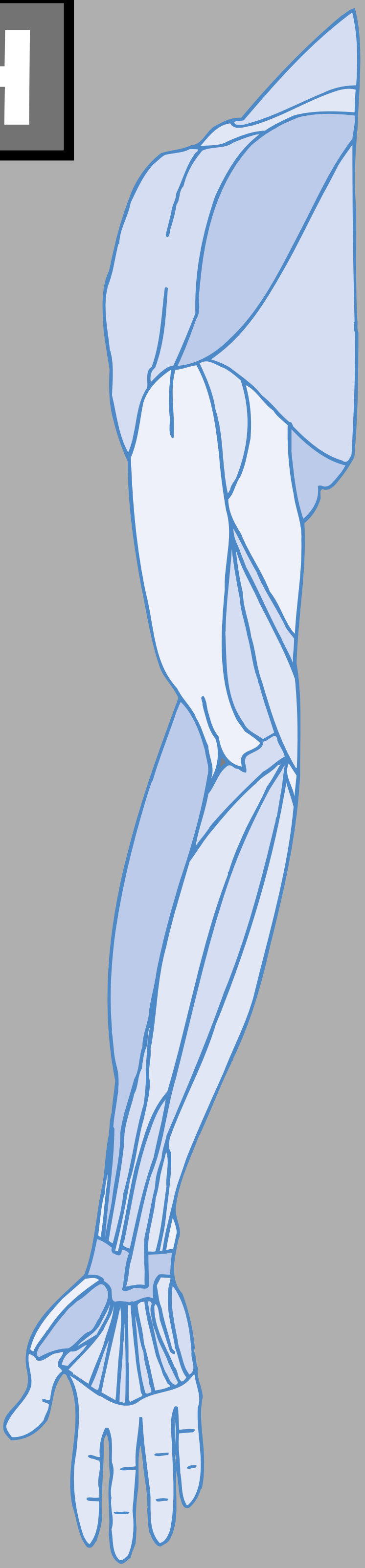

INSTRUCTIONS

This questionnaire asks about your symptoms as well as your ability to perform certain activities.

Please answer *every question*, based on your condition in the last week, by circling the appropriate number.

If you did not have the opportunity to perform an activity in the past week, please make your *best estimate* on which response would be the most accurate.

It doesn't matter which hand or arm you use to perform the activity; please answer based on your ability regardless of how you perform the task.

Disabilities of the Arm, Shoulder and Hand

Please rate your ability to do the following activities **during the week before the accident**

	NO DIFFICULTY	MILD DIFFICULTY	MODERATE DIFFICULTY	SEVERE DIFFICULTY	UNABLE
1. Open a tight or new jar.	1	2	3	4	5
2. Write.	1	2	3	4	5
3. Turn a key.	1	2	3	4	5
4. Prepare a meal.	1	2	3	4	5
5. Push open a heavy door.	1	2	3	4	5
6. Place an object on a shelf above your head.	1	2	3	4	5
7. Do heavy household chores (e.g., wash walls, wash floors).	1	2	3	4	5
8. Garden or do yard work.	1	2	3	4	5
9. Make a bed.	1	2	3	4	5
10. Carry a shopping bag or briefcase.	1	2	3	4	5
11. Carry a heavy object (over 10 lbs).	1	2	3	4	5
12. Change a lightbulb overhead.	1	2	3	4	5
13. Wash or blow dry your hair.	1	2	3	4	5
14. Wash your back.	1	2	3	4	5
15. Put on a pullover sweater.	1	2	3	4	5
16. Use a knife to cut food.	1	2	3	4	5
17. Recreational activities which require little effort (e.g., cardplaying, knitting, etc.).	1	2	3	4	5
18. Recreational activities in which you take some force or impact through your arm, shoulder or hand (e.g., golf, hammering, tennis, etc.).	1	2	3	4	5
19. Recreational activities in which you move your arm freely (e.g., playing frisbee, badminton, etc.).	1	2	3	4	5
20. Manage transportation needs (getting from one place to another).	1	2	3	4	5
21. Sexual activities.	1	2	3	4	5

DISABILITIES OF THE ARM, SHOULDER AND HAND

	NOT AT ALL	SLIGHTLY	MODERATELY	QUITE A BIT	EXTREMELY
22. During the past week, *to what extent* has your arm, shoulder or hand problem interfered with your normal social activities with family, friends, neighbours or groups? *(circle number)*	1	2	3	4	5

	NOT LIMITED AT ALL	SLIGHTLY LIMITED	MODERATELY LIMITED	VERY LIMITED	UNABLE
23. During the past week, were you limited in your work or other regular daily activities as a result of your arm, shoulder or hand problem? *(circle number)*	1	2	3	4	5

Please rate the severity of the following symptoms in the last week. *(circle number)*

	NONE	MILD	MODERATE	SEVERE	EXTREME
24. Arm, shoulder or hand pain.	1	2	3	4	5
25. Arm, shoulder or hand pain when you performed any specific activity.	1	2	3	4	5
26. Tingling (pins and needles) in your arm, shoulder or hand.	1	2	3	4	5
27. Weakness in your arm, shoulder or hand.	1	2	3	4	5
28. Stiffness in your arm, shoulder or hand.	1	2	3	4	5

	NO DIFFICULTY	MILD DIFFICULTY	MODERATE DIFFICULTY	SEVERE DIFFICULTY	SO MUCH DIFFICULTY THAT I CAN'T SLEEP
29. During the past week, how much difficulty have you had sleeping because of the pain in your arm, shoulder or hand? *(circle number)*	1	2	3	4	5

	STRONGLY DISAGREE	DISAGREE	NEITHER AGREE NOR DISAGREE	AGREE	STRONGLY AGREE
30. I feel less capable, less confident or less useful because of my arm, shoulder or hand problem. *(circle number)*	1	2	3	4	5

DASH DISABILITY/SYMPTOM SCORE = _____ ([(sum of n responses / n) - 1] x 25, where n is the number of completed responses.)

A DASH score may <u>not</u> be calculated if there are greater than 3 missing items.

DISABILITIES OF THE ARM, SHOULDER AND HAND

WORK MODULE (OPTIONAL)

The following questions ask about the impact of your arm, shoulder or hand problem on your ability to work (including homemaking if that is your main work role).

Please indicate what your job/work is:____

❐ I do not work. (You may skip this section.)

Please circle the number that best describes your physical ability in the past week. Did you have any difficulty:

	NO DIFFICULTY	MILD DIFFICULTY	MODERATE DIFFICULTY	SEVERE DIFFICULTY	UNABLE
1. using your usual technique for your work?	1	2	3	4	5
2. doing your usual work because of arm, shoulder or hand pain?	1	2	3	4	5
3. doing your work as well as you would like?	1	2	3	4	5
4. spending your usual amount of time doing your work?	1	2	3	4	5

SPORTS/PERFORMING ARTS MODULE (OPTIONAL)

The following questions relate to the impact of your arm, shoulder or hand problem on playing *your musical instrument or sport or both*.

If you play more than one sport or instrument (or play both), please answer with respect to that activity which is most important to you.

Please indicate the sport or instrument which is most important to you:______________________________

❐ I do not play a sport or an instrument. (You may skip this section.)

Please circle the number that best describes your physical ability in the past week. Did you have any difficulty:

	NO DIFFICULTY	MILD DIFFICULTY	MODERATE DIFFICULTY	SEVERE DIFFICULTY	UNABLE
1. using your usual technique for playing your instrument or sport?	1	2	3	4	5
2. playing your musical instrument or sport because of arm, shoulder or hand pain?	1	2	3	4	5
3. playing your musical instrument or sport as well as you would like?	1	2	3	4	5
4. spending your usual amount of time practising or playing your instrument or sport?	1	2	3	4	5

SCORING THE OPTIONAL MODULES: Add up assigned values for each response; divide by 4 (number of items); subtract 1; multiply by 25.

An optional module score may <u>not</u> be calculated if there are any missing items.

Health Questionnaire (EQ-5D-5L)

Under each heading, please tick the ONE box that best describes your health
one week before the accident

MOBILITY

- ☐1 I have no problems in walking about
- ☐2 I have slight problems in walking about
- ☐3 I have moderate problems in walking about
- ☐4 I have severe problems in walking about
- ☐5 I am unable to walk about

SELF-CARE

- ☐1 I have no problems washing or dressing myself
- ☐2 I have slight problems washing or dressing myself
- ☐3 I have moderate problems washing or dressing myself
- ☐4 I have severe problems washing or dressing myself
- ☐5 I am unable to wash or dress myself

USUAL ACTIVITIES *(e.g. work, study, housework, family or leisure activities)*

- ☐1 I have no problems doing my usual activities
- ☐2 I have slight problems doing my usual activities
- ☐3 I have moderate problems doing my usual activities
- ☐4 I have severe problems doing my usual activities
- ☐5 I am unable to do my usual activities

PAIN / DISCOMFORT

- ☐1 I have no pain or discomfort
- ☐2 I have slight pain or discomfort
- ☐3 I have moderate pain or discomfort
- ☐4 I have severe pain or discomfort
- ☐5 I have extreme pain or discomfort

ANXIETY / DEPRESSION

- ☐1 I am not anxious or depressed
- ☐2 I am slightly anxious or depressed
- ☐3 I am moderately anxious or depressed
- ☐4 I am severely anxious or depressed
- ☐5 I am extremely anxious or depressed

UK (English) © 2009 EuroQol Group EQ-5D™ is a trade mark of the EuroQol Group

Health Questionnaire (EQ-5D-5L)

- We would like to know how good or bad your health is
- This scale is numbered from 0 to 100.
- 100 means the <u>best</u> health you can imagine.
  0 means the <u>worst</u> health you can imagine.
- Mark an X on the scale to indicate how your health is
- Now, please write the number you marked on the scale in the

  below.

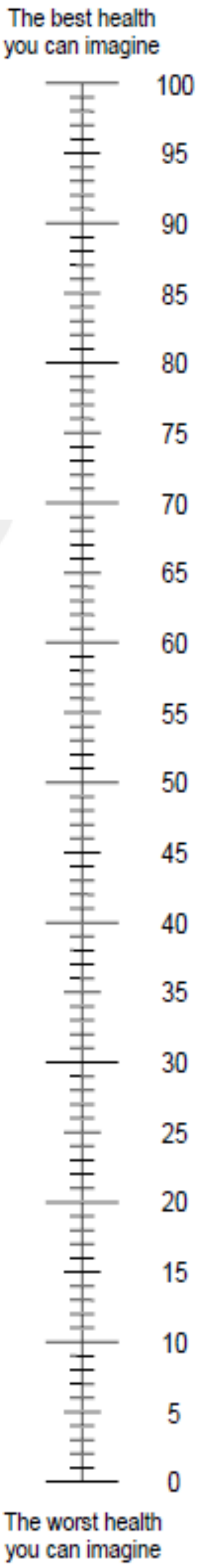

YOUR HEALTH

UK (English) © 2009 EuroQol Group EQ-5D™ is a trade mark of the EuroQol Group
