# Supplementary material for: A natural experiment study: Low-profile double plating versus single plating techniques in midshaft clavicle fractures—Study protocol
Source: PLoS One. 2023 Sep 8;18(9):e0291238. doi: 10.1371/journal.pone.0291238 (PMC10490911; doi:10.1371/journal.pone.0291238)
Supplement: S6 File — (PDF) [file pone.0291238.s006.pdf]

A natural experiment study: Low-profile double plating versus single plating techniques in midshaft clavicle fractures - Study protocol

Yannic Lecoultre[1,2]*, Bryan JM van de Wall[1,2,5], Nadine Diwersi[1,5], Steffen W Pfarr[3], Beat Galliker[4], Reto Babst[1,2], Björn-Christian Link[1], Frank JP Beeres[1]

1. Lucerne Cantonal Hospital, Department of Orthopedic and Trauma Surgery, Spitalstrasse, 6000 Luzern 16, Switzerland

2. University of Lucerne, Faculty of Health Sciences and Medicine, Frohburgstrasse 3, 6002 Luzern, Switzerland

3. Schwyz Hospital, Department of Surgery, Waldeggstrasse 10, 6430 Schwyz, Switzerland

4. Sursee Hospital, Department of Surgery, Spitalstrasse 16A, 6210 Sursee, Switzerland

5. Obwalden Cantonal Hospital, Department of Surgery, Brünigstrasse 181, 6060 Sarnen, Switzerland

* Corresponding Author

E-mail: yannic.lecoultre@luks.ch

Abstract

Background

Single plate osteosynthesis is commonly employed when performing surgical stabilization of midshaft clavicle fractures. In recent years, a smaller structural low-profile double plating technique has been described as a possible solution for the high removal rates associated with single plating. A previous meta-analysis has demonstrated that low-profile double plating attains the same healing rates as single plating without a higher chance of fracture-related infections. This meta-analysis, however, was based on relatively small studies. Therefore, a multicentre prospective natural experiment was designed using natural variation in treatment regimens and geographical location of the trauma as treatment allocation mechanism to compare both treatments on a larger scale. This manuscript describes its protocol.

Material & Methods

Patients (≥18 years) with primary midshaft clavicle fractures that are eligible for operative treatment will be included. Treatment allocation will be determined by the geographical location of the accident and local hospital providing treatment. In two centres, single plating is the treatment of choice for these patients. In two others, low-profile double plating has become the standard treatment. For the low-profile double plating group, one superiorly positioned VariAx 2.0mm and one anterior VariAx 2.4mm or 2.7mm plate will be used. For the single plating group, the standard locally available implant will be used. A total of 336 patients will be included. The primary outcome of interest is re-intervention. Secondary outcomes include complications, operative time, length of incision, functional scores (DASH, EQ-5D-DL, VAS-Pain/Satisfaction) and cost-effectiveness.

Discussion

This study will determine whether low-profile double plating has significant clinical and cost-effective benefits over single plating techniques in midshaft clavicle fractures. The study will also give insight in the performance of a natural experiment study design for orthopedic trauma research.

Trial registration

This study has been registered on ClincialTrials.gov, identifier NCT 05579873.

Keywords

Clavicle injuries, Bone Plates, Non-randomized Controlled Trials as Topic, Natural Experiments

Background

Exposed to the exterior environment due to a lack of surrounding muscles or subcutaneous tissue, the clavicle is prone to traumatic injury. Clavicle fractures account for 5 to 6% of all fractures, of which 69 to 81% occur in the midshaft. [1,2] Surgical stabilization using a single plate, positioned either anteriorly or superiorly, is the most common surgical technique when patients/surgeons decide on operative treatment. [3] The major disadvantage of plate fixation, however, is implant irritation. As a result, up to 64% of patients undergo re-interventional surgery for implant removal. [4]

In recent years a low-profile double plating technique with smaller plates has been described as a possible solution to reduce the high rates of implant irritation. [5] Biomechanically, low-profile double plating is expected to be at least as stable as single plating. [6] A recently published meta-analysis showed that low-profile double plating for midshaft clavicle fractures is a safe procedure attaining the same high union rates seen in patients treated with single plating. [7] Additionally, double plating appears to have a lower overall complication and reintervention rate, mainly due to the lower incidence of implant-related complaints. Low-profile dual plating, however, is a relatively new technique and should be further explored to test whether our findings can be confirmed.

Although randomized controlled trials (RCTs) are considered the gold standard for testing the effectiveness of such new interventions, the artificial conditions that are usually imposed on surgical practice frequently limit the feasibility and the generalizability of its results. A natural experiment (NE) design offers a possible solution. NEs are observational studies in which patients are exposed to either the experimental or the control condition, and treatment allocation is determined by factors outside the control of the investigators (e.g., geographical location). [8] Although not the same, the process governing treatment allocation arguably resembles that of randomization, since treatment allocation is expected to be (to a large extent) independent of patients' characteristics and their prognosis.

This protocol encompasses a prospective NE comparing low-profile double plating to single plating in midshaft clavicle fractures. The primary endpoint is the overall re-intervention rate, with a specific interest in implant removal due to irritation. Secondary endpoints include all complications, healing, the general quality of life, functional results, and cost-effectiveness.

Methods/Design

Aim

To compare single plating to low-profile double plating in midshaft clavicle fractures with regard to re-intervention, complications, healing, quality of life, functional results, and cost-effectiveness.

Study design

This study will be a multicenter prospective cohort study between four Swiss centers with comparable patient case-mix. At which hospital/surgeon trauma patients are presented after an accident depends on the geographical location of the accident. We hypothesize that different Swiss centers treat a similar caseload of trauma patients with clavicle fracture and that the patient case-mix is similar across the different centers.

Of the four participating centers, two centers are specialized in single plating, and two are specialised in low-profile double plating will participate. Treatments will be naturally allocated by the geographical location of the accident. Although centers are divided into either category based on their standard treatment of care, surgeons will be allowed to alter their treatment plan based on their own surgical expertise and according to what they (after consultation of the patient) consider the most appropriate treatment. This study design is sometimes referred to as a NE and is arguably less prone to confounding bias than conventional observational studies, provided the above-mentioned assumptions hold.

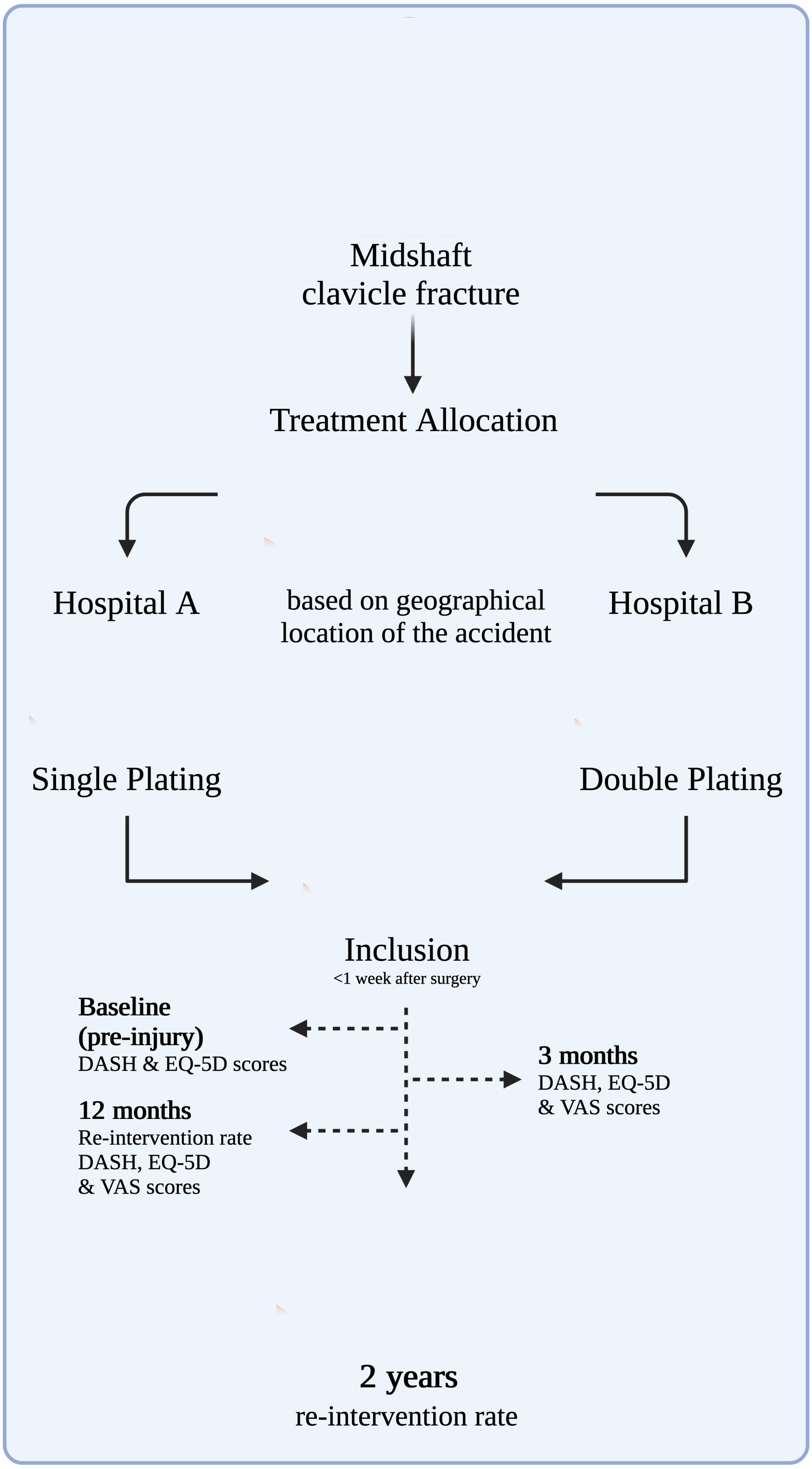

Figure 1 Schedule of enrolment, interventions, and assessments

Participant selection

Eligibility criteria

All adult patients (>18 older) presenting at the emergency department (ED) or outpatient clinic with midshaft clavicle fractures (Robinson Type II or AO/OTA 15.2) [9] will be included. In-and exclusion criteria are shown in Figure 2. Patients will be screened for eligibility by the treating physician at presentation to the emergency department or during outpatient visits (within two weeks after trauma).

INCLUSION CRITERIA

- Aged 18 years or older.
- Written informed consent.
- Primary midshaft clavicle fracture (Robinson Type II or AO 15.2).
- Patients that are eligible for operative treatment of clavicle fracture, which may include but is not limited by:
  - Displacement of one or more shaft width.
  - Shortening of more than 1cm in length.
  - High physicial activity level.

EXCLUSION CRITERIA

- Delayed presentation (>14 days).
- Initial operative treatment at a non-participating hospital.
- Open fractures.
- Pathological fractures.
- Re-fractures of the clavicle.
- Cognitive impairment or language barrier precluding answering questionnaires.
- Unable to complete follow-up (e.g. different residential area/tourist).

Figure 2 In/Exclusion criteria

Participant recruitment and screening

Patients will be asked to participate in the study after the established decision on operative treatment. If eligible, the treating physician or local investigator will explain to each participant the nature of the study, its purpose, the procedures involved, the expected duration, the potential risks and benefits, and any discomfort it may entail. Each participant will be informed that participation in the study is voluntary, that he or she may withdraw from the study at any time, and that withdrawal of consent will not affect his or her subsequent medical assistance and treatment. The participant will be informed that their pseudonymized medical records may be examined by authorized researchers other than their treating physician.

Data management

Data will be stored in pseudonymized form in an encrypted online database by the local investigators, which can access their respective individual patient data. Full access to the database is granted to the study coordinator.

Study interventions

Low-profile double plating group

Low-profile double plating consists of one Stryker VariAx 2.0mm plate positioned on the superior aspect of the clavicle and a second Stryker VariAx 2.4mm or 2.7mm on the anterior side. Use of this implant will be according to the manufacturer's indication of use.

Single plating group

The choice of implant used for single plating is left at the discretion of the treating surgeon and the availability of implants in participating centers. Possible implants consist of 2.7 and 3.5mm plates placed on the superior or anterior side.

Standardization of care

Preoperative care will consist of general anesthesia and routine antibiotic prophylaxis. Incision and cutaneous nerve sparing are left at the treating surgeon's discretion and will be mentioned in the operation report. In case of a multi-fragmentary fracture pattern, bridge plating will be performed. In case of a simple fracture, a neutralization plate (with or without lag screws) or a compression plate will be used. Closure of deltopectoral fascia with absorbable braided sutures and skin with absorbable or nonabsorbable monofilament sutures will be performed for all patients. Operations will either be performed or directly supervised by a specialized trauma surgeon. Aftercare comprises a sling for comfort for 1-2 weeks, no weight bearing for six weeks, and outpatient clinic visits, including AP and axial clavicle X-rays at six weeks, three months, and one year after surgery in both treatment groups.

Study outcomes

The primary outcome of this study is the number of re-interventions after two years of follow-up. Secondary outcomes include the number of re-interventions at one year of follow-up, all complications such as fracture-related infection [10], (a)symptomatic non-union, numbness below the scar, and self-reported implant irritations, disability of the shoulder and arm (DASH) score, EuroQol (EQ)-5D score, and the visual analogue scale (VAS) score for pain and patient satisfaction at 3, 12, and 24 months follow-up. A complete overview of the primary and secondary outcomes can be found in Table 1.

**Primary Objective**
Re-intervention (all indications) after 2-year follow-up
**Secondary Objective**
Re-intervention rate (including implant removal) after 1-year follow-up.

All other complications (including complications treated non-operatively)

- Fracture related infection.
- Symptomatic non-union defined as absence of radiological signs of healing (callus formation or fading of fractures lines) combined with pain at the fracture site at 9 months.
- Asymptomatic non-union defined as absence of radiological signs of healing (callus formation or fading of fracture lines) without any clinical symptoms.
- Numbness below scar line related postoperatively and at 12-months follow-up.
- Self-reported implant irritation or other reasons for implant removal at 12 months.

Operative time.

Length of surgical incision.

QuickDASH score at baseline (pre-injury), 3- and 12-months follow-up.

EQ-5D-5L at baseline (pre-injury), 3- and 12-months follow-up.

VAS pain and patient satisfaction at 3- and 12-months follow-up.

Table 1: Primary and secondary outcomes.

The outcome measure, follow-up and timeline

At baseline, the treating physician or local investigator will collect the following characteristics: age, gender, body mass index (BMI), smoking status, comorbidities, medication, trauma mechanism, concomitant injuries and planned operative procedure (including treating hospital and surgeon), pre-injury DASH and EQ-5D-5L. The x-ray at presentation or outpatient clinic visit will be used to determine fracture type and dislocation. Fracture type will be determined according to the AO/OTA classification system for midshaft clavicle fractures. [9] Patients will receive the operation within two weeks of presentation.

The patients will return to the outpatient clinic at three and twelve months. The outpatient clinic visit will include several questionnaires (e.g., VAS, EQ5D, DASH), x-rays, and a physical examination (Table 2). All data and adverse events (AE) will be registered. At two years, the patient will be either

seen at the outpatient clinic visit or interviewed by telephone, and the electronic patient database will be accessed to assess patient-reported outcome measures (PROMS), complications and reintervention rate.

All the measurements and examinations described are part of standard care, except the questionnaires. The questionnaires will be filled in at regular outpatient clinic visits or by telephone interview.

	Baseline (at in-lcusion)	12-weeks follow-up	12-months follow-up	24-months follow-up
Re-intervention			X	X
X-ray	X	X	X	
DASH*	X	X	X	
EQ5D-5L*	X	X	X	
VAS pain and satisfaction*	X	X	X	
Complications		X	X	X

*** Not part of standard care.**

Table 2. Outcome measures and planning

Sample size calculation

The sample size calculation is based on the primary outcome (re-intervention). Based on a recent meta-analysis of our study group, a risk reduction of 10% is expected in favor of low-profile double plating compared to single plating. To demonstrate this difference using a chi-square-test (alpha =

0.05, power = 0.8) approximately 152 patients per treatment group are needed. To compensate for a potential 5% loss to follow-up, a total of 336 patients are required.

Statistical analysis.

The statistical package IBM SPSS Statistics for Windows, version 28.0, will be used for analysis. Depending on the normality distribution, baseline characteristics will be described as means and standard deviations or median and interquartile range for continuous variables. Categorical variables will be reported in counts and percentages. Differences between treatment groups will be analyzed with an independent samples student's T-test or Mann-Whitney-U test (depending on distribution). Categorical variables will be analyzed using the Chi-square test.

The primary outcome will be analyzed using logistic regression with revision surgery as the dependent and treatment as the independent variable. The relative risk (RR) will be calculated with a 95% confidence interval (95% CI). Multivariable logistics regression analysis will also be performed to account for potential known confounders including age and fracture type.

Missing data (caused by incomplete hospital documentation or loss-to-follow-up) will be addressed using multiple imputation techniques.

Discussion

Previously, a meta-analysis on single vs low-profile double plating demonstrated that low-profile double plating attains the same healing rates as single plating without a higher chance of fracture-related infections. [7] This study however, was based on studies with relatively small sample sizes. The present NE study will provide the required evidence to confirm or revoke the aforementioned conclusions based on a more extensive study population.

Additionally, this study will form the basis for a more prominent use of the NE design in the surgical research. Due to its design, this study has a high feasibility with minimal burden to the participants, since the received care is that of daily clinical practice. Not only will the study help us understand if low-profile double plating has significant clinical and cost-effective benefits over single plating techniques in midshaft clavicle fractures, but it also provides new insights into how the NE design functions in a research field where it has rarely been used before.

List of abbreviations

AE	Adverse Event
BMI	Body MASS Index
CI	Confidence Interval
DASH	Disabilities of the arm, shoulder, and hand
EQ	EuroQol
NE	Natural Experiment
OR	Odds Ratio
RCT	Randomized Controlled Trial

Ethics approval and consent to participate

This study was ethically approved by the Northwestern and central Swiss ethics commission. The identifier number is 2022-00574

Publication

Publications based on the results obtained shall not be made before the first multicentre publication or presentation, which shall be coordinated by the principal investigator unless otherwise agreed in writing. The manuscript shall be published in a peer-reviewed journal.

Consent for publication

Not applicable

Availability of data and materials

The datasets used and/or analyzed during the current study are available from the corresponding author upon reasonable request.

Author's contributions

Conceptualization: F.B and B.W.

Methodology: F.B, B.W, Y.L.

Investigation: Y.L, S.P and B.G.

Writing - Original Draft Preparation: N.D, Y.L, B.W and F.B.

Writing - Review and Editing: F.B, B.L, R.B.

Project administration: Y.L.

Funding acquisition: F.B, B.W.

Acknowledgments

Not applicable

Protocol Amendments

Relevant protocol changes (eg changes to eligibility criteria, outcomes, analyses) will be discussed within the research group and have to be approved by the trial registries, the Northwestern and central Swiss ethics commission and the sponsor.

References

1. van der Meijden OA, Gaskill TR, Millett PJ. Treatment of clavicle fractures: current concepts review. J shoulder Elb Surg. 2012;21: 423–429. doi:10.1016/j.jse.2011.08.053
2. Postacchini F, Gumina S, De Santis P, Albo F. Epidemiology of clavicle fractures. J shoulder Elb Surg. 2002;11: 452–456. doi:10.1067/mse.2002.126613
3. Wiesel B, Nagda S, Mehta S, Churchill R. Management of Midshaft Clavicle Fractures in Adults. J Am Acad Orthop Surg. 2018;26: e468–e476. doi:10.5435/JAAOS-D-17-00442
4. Wijdicks FJG, Millett PJ, Houwert RM, Van Der Meijden OAJ, Verleisdonk EJMM. Systematic review of the complications of plate fixation of clavicle fractures. Arch Orthop Trauma Surg. 2012;132: 617–625. doi:10.1007/s00402-011-1456-5
5. Chen MJ, DeBaun MR, Salazar BP, Lai C, Bishop JA, Gardner MJ. Safety and efficacy of using 2.4/2.4 mm and 2.0/2.4 mm dual mini-fragment plate combinations for fixation of displaced diaphyseal clavicle fractures. Injury. 2020;51: 647–650. doi:10.1016/j.injury.2020.01.014
6. Zhang F, Chen F, Qi Y, Qian Z, Ni S, Zhong Z, et al. Finite element analysis of dual small plate fixation and single plate fixation for treatment of midshaft clavicle fractures. J Orthop Surg Res. 2020;15: 148. doi:10.1186/s13018-020-01666-x
7. Rompen IF, van de Wall BJM, van Heijl M, Bünter I, Diwersi N, Tillmann F, et al. Low profile dual plating for mid-shaft clavicle fractures: a meta-analysis and systematic review of observational studies. Eur J Trauma Emerg Surg. 2022;48: 3063–3071. doi:10.1007/s00068-021-01845-3
8. Leatherdale ST. Natural experiment methodology for research: a review of how different methods can support real-world research. Int J Soc Res Methodol. 2019;22: 19–35. doi:10.1080/13645579.2018.1488449
9. Meinberg EG, Agel J, Roberts CS, Karam MD, Kellam JF. Fracture and Dislocation Classification Compendium-2018. J Orthop Trauma. 2018;32 Suppl 1: S1–S170. doi:10.1097/BOT.0000000000001063
10. Metsemakers WJ, Morgenstern M, McNally MA, Moriarty TF, McFadyen I, Scarborough M, et al. Fracture-related infection: A consensus on definition from an international expert group. Injury. 2018;49: 505–510. doi:10.1016/j.injury.2017.08.040
