# Supplementary material for: A natural experiment study: Low-profile double plating versus single plating techniques in midshaft clavicle fractures—Study protocol
Source: PLoS One. 2023 Sep 8;18(9):e0291238. doi: 10.1371/journal.pone.0291238 (PMC10490911; doi:10.1371/journal.pone.0291238)
Supplement: Attachment — Submitted filename: Response to Reviewers.docx [file pone.0291238.s007.docx]

**Response to Reviewers**

**Journal requirements**

When submitting your revision, we need you to address these additional requirements.

1. Please ensure that your manuscript meets PLOS ONE's style requirements, including those for file naming. The PLOS ONE style templates can be found at

<https://journals.plos.org/plosone/s/file?id=wjVg/PLOSOne_formatting_sample_main_body.pdf> and <https://journals.plos.org/plosone/s/file?id=ba62/PLOSOne_formatting_sample_title_authors_affiliations.pdf>

*We have re-checked PLOS One's style requirements and adapted our titlepage, formatting, figure captions and file naming.*

2. Your ethics statement should only appear in the Methods section of your manuscript. If your ethics statement is written in any section besides the Methods, please move it to the Methods section and delete it from any other section. Please ensure that your ethics statement is included in your manuscript, as the ethics statement entered into the online submission form will not be published alongside your manuscript.

*We have moved the ethics statement to the methods section.*

3. We note that the original protocol that you have uploaded as a Supporting Information file contains an institutional logo. As this logo is likely copyrighted, we ask that you please remove it from this file and upload an updated version upon resubmission.

*Thank you for the notice, we removed all institutional logos from the supporting information files.*

4. Please review your reference list to ensure that it is complete and correct. If you have cited papers that have been retracted, please include the rationale for doing so in the manuscript text, or remove these references and replace them with relevant current references. Any changes to the reference list should be mentioned in the rebuttal letter that accompanies your revised manuscript. If you need to cite a retracted article, indicate the article’s retracted status in the References list and also include a citation and full reference for the retraction notice.

*We have re-checked our reference list and replaced reference nr. 8, since it is not listed in medline. Also, we added reference 11 and 12 to meet reviewer 2's requirements.*

**Comments to the Author**

Reviewer #1: please give the clear definition of the terms: re-interventions, healing and complications?
How exactly are these terms defined at what point in time? What influence do they have on fracture healing and why? How long is the follow-up?
In addition, inclusion and exclusion criteria are not known. What about open fractures? Polytrauma patients? pre-existing conditions? infections? Osteoporosis? Infection as a cause of non-healing?
Duration of surgery? Status of the operator? Number of screws used? Time of accident to operation? Postoperative procedure? Will this be the same for all? immobilization? Suture technique? (Intracutaneous vs. donati?)
Fracture classification?
there also needs to be a clear definition of when the fracture is considered healed and how this is determined and when.

*Thank you for your detailed and valuable feedback. We fully agree with your comments. The inclusion criteria, outcome definitions, timepoint of outcome measurement and follow-up duration are described in figures 1, 2 and table 1. We also added a section on operative data that will be collected after the surgical procedure and a description of fracture classification used. Please let us know if you have any additional wishes.*

Reviewer #2: 1) General comment
The authors would like to show in their study protocoll “Low-profile double plating versus single plating techniques in midshaft clavicule fractures” clinical and cost-effective benefits between double plating and single plating techniques. They also are trying to gibe insights in the performance of a natural experiment study design for orthopedic trauma research..
The study design is very well thought out or worked out for the questions mentioned. However, it is worth discussing whether the phase of drafting a study design is the right time to publish. At the current time, an infinite number of works that have been carried out are being published. The entire field of medicine is flooded with publications, so that there is not enough time to evaluate the relevant publications. If every protocol of future studies is published now, the flood of publications will only increase. Ultimately, the protocols have not yet proven any essential facts regarding innovations, new therapeutic approaches or new findings.
In any case, it is interesting whether or not the double plating technique has any really significant clinical and cost-effective advantages over the single plating application. This study can certainly be carried out in the setting mentioned and will show corresponding well-founded results. However, it is necessary to wait until the results of the content are available before one wants to expect this from a broader readership. However, if one publishes future study protocols, this will lead to an infinitely progressive increase in publications
If you look at the most famous collection of scientific reports, namely PubMed, around 500,000 documents are published there every year. This entire medical collection is growing so rapidly that one should focus on essential results. This database now includes more than 32 million "citations" from scientific journal articles and online literature if you only look at the biomedical literature (PubMed Help).

2) Specific suggestions
The study mentioned above is well designed and will be appropriate for the intended research question and outcome results

*First, we want to thank you for your appreciative feedback on our study approach. We agree with you that the public libraries are being flooded with publications. The quality of these studies also varies considerably. This is however precisely the reason why we think it is important to publish protocols prior to conducting the study itself including those from prospective observational studies. This creates transparency on the study design making it easier for readers to assess the quality and validity of the end results; a feature many publications are lacking. We agree that the number of publications should be reduced in such a way that only good quality data finds its way to our public libraries. Protocols, in our opinion, are the gatekeepers for quality and should not be seen as just an additional publication but rather a sign or mark of quality when the end results do appear. We hope you may understand our point of view and support the publication of this protocol.*

Reviewer #3: In this study protocol, a non-randomized multicenter prospective clinical trial is being proposed to determine if low-profile double plating shows significant clinical and cost-effective benefits in comparison to single plating techniques. The primary outcome is re-intervention rate. Secondary outcomes are complications, operative time, length of incision, function scores, and cost-effectiveness. The target sample size is 336.

Minor revisions:
1- Table at line 158: inclusion is misspelled.

*Thank you for pointing this out. We corrected this mistake.*

2- Line 167: Indicate if the alpha level was one or two-sided. A single sided test seems appropriate since the low-profile double plating is expected to outperform the single plating technique.

*This was a two-sided alpha level. Indeed, based on prior studies we expect that the reintervention rate will be lower among the double plating group. A one-sided test would theoretically be correct. However, we are not absolutely certain this will be the case. For this reason, we prefer to play it safe and taking this uncertainty into account by using a two-sided alpha in our sample size calculation.*

3- For outcomes that are repeatedly measured, statistical methods appropriate for analyzing repeated measures should be used.

*Thank you for the input, we now specified this in the statistical analysis section.*
